# Brain-transportable dipeptides across the blood-brain barrier in mice

**DOI:** 10.1038/s41598-019-42099-9

**Published:** 2019-04-08

**Authors:** Mitsuru Tanaka, Shinya Dohgu, Genki Komabayashi, Hayato Kiyohara, Fuyuko Takata, Yasufumi Kataoka, Takashi Nirasawa, Motohiro Maebuchi, Toshiro Matsui

**Affiliations:** 1 Department of Bioscience and Biotechnology, Faculty of Agriculture, Graduate School of Kyushu University, 744 Motooka, Nishi-ku, Fukuoka, 819-0395 Japan; 2 Department of Pharmaceutical Care and Health Sciences, Faculty of Pharmaceutical Sciences, Fukuoka University, 8-19-1 Nanakuma, Jonan-ku, Fukuoka, 814-0180 Japan; 3 Bruker Japan K.K., 3-9 Moriya-cho, Kanagawa-ku, Yokohama, Kanagawa 221-0022 Japan; 4 FUJI OIL CO., Ltd., Research and Development Division, 1 Sumiyoshi-cho, Izumisano, Osaka, 598-8540 Japan

## Abstract

Apart from nutrients required for the brain, there has been no report that naturally occurring peptides can cross the blood-brain barrier (BBB). The aim of this study was to identify the BBB-transportable peptides using *in situ* mouse perfusion experiments. Based on the structural features of Gly-*N*-methylated Gly (Gly-Sar), a reported BBB-transportable compound, 18 dipeptides were synthesized, and were perfused in the mouse brain for two minutes. Among the synthesized dipeptides, Gly-Sar, Gly-Pro, and Tyr-Pro were transported across the BBB with *K*_*i*_ values of 7.60 ± 1.29, 3.49 ± 0.66, and 3.53 ± 0.74 µL/g·min, respectively, and accumulated in the mouse brain parenchyma. Additionally, using MALDI-MS/MS imaging analysis of Tyr-Pro-perfused brain, we provide evidence for Tyr-Pro accumulation in the hippocampus, hypothalamus, striatum, cerebral cortex, and cerebellum of mouse brain.

## Introduction

The physiological preference of peptide uptake has been demonstrated in human and animal studies. In subjects with mild hypertension the intake of a dipeptide, Val-Tyr, for a month modulated blood pressure^1^. The dipeptide, Trp-His showed an apparent anti-atherosclerotic effect in apo E-deficient mice, while no effect was observed on administrating a mixture of Trp and His^2^. We also demonstrated that the aforementioned bioactive peptides are absorbed in their intact form across intestinal membrane into blood^3,4^. Peptide transport across the epithelial layer in animal organs is mainly regulated by diverse proton-coupled oligopeptide transporters of the SLC15 family, which have a selective preference for di-/tripeptides^5^. Peptide transporter 1 (PepT1) in the intestine and PepT2 in the kidney are known to be involved in the absorption and reabsorption of di-/tripeptides^6,7^. Thus far, it has been reported that transporters for peptide incorporation are expressed in multiple organs such as the liver, blood vessels, muscle, brain, intestine, and kidney. However, no studies report the intact absorption of dipeptides beyond the blood-brain barrier (BBB).

It is well known that the brain selectively absorbs useful food compounds such as amino acids, glucose, inorganic compounds, and vitamins, but tightly inhibits the uptake of any other substrates, since the BBB can limit paracellular permeation by providing a meshwork of non-fenestrated microvessel endothelial cells surrounded by pericytes, astrocytes and neurons^8^. Although it has been reported that cell penetrating peptides (CPP) mostly composed of >10 amino acids possessing cationic, amphipathic or hydrophobic properties can enter the central nervous system (CNS) by energy-independent passive penetration or energy-dependent endocytosis penetration pathways^9^, there are no studies showing the intact transport of small hydrophilic peptides (i.e., di-/tripeptides) beyond the BBB system. In the BBB system, the transporters of PepT2 and peptide/histidine transporter 1 (PHT1), but not PepT1, were expressed at the blood-cerebrospinal fluid (CSF)-barrier composed of the choroid plexus epithelium^7^. The brain PepT2 may play a role in pumping out metabolites produced from endogenous neuronal dipeptides for their clearance from the CSF^7,10^. An *in vivo* pharmacokinetic study using *Pht1*-null mice^11^ revealed that PHT1 is expressed throughout the brain including choroid plexus, and preferably recognizes histidines. Although Hu *et al*.^12^ also studied Gly-*N*-methylated Gly (Gly-Sar) uptake into the brain through PHT1 in adult rodents, information on brain PHT1 substrates is limited. However, recent *in vivo* findings on the improvement of delayed memory score in patients with mild cognitive impairment^13^ and on the suppression of cognitive decline induced by neurotrophic factors in SAMP8 mice by a diet containing di-/tripeptides^14^ strongly led us to speculate the possible intact transport of di-/tripeptides beyond the BBB.

Thus, in the present *in situ* mouse brain perfusion study, we demonstrate that small peptides (in this study, dipeptides) can be transported across the BBB and accumulate in the brain. We also performed a mass spectrometry (MS)-visualization experiment with phytic acid-aided matrix assisted laser desorption ionization (MALDI)–MS/MS imaging^15^ to provide a direct evidence of the accumulation of dipeptides in the brain.

## Results

### Identification of dipeptides capable of intact transport across the mouse BBB

**Figure 1:**
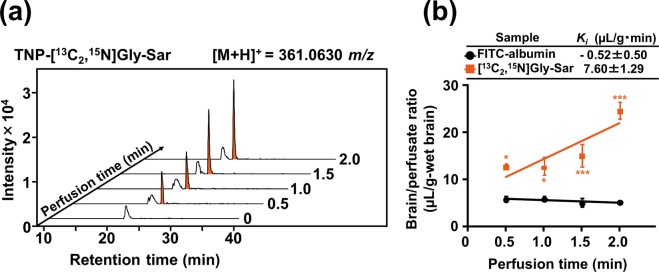
Transport of [^13^C_2_,^15^N]Gly-Sar across ICR mouse BBB post *in situ* brain perfusion. (**a**) Stacked extracted ion chromatograms of TNP-[^13^C_2_,^15^N]Gly-Sar ([M + H]^+^, 361.0630 *m*/*z*) perfused brain at time intervals of 0, 0.5, 1.0, 1.5, and 2.0 min, respectively, as examined by LC-TOF/MS. TNBS derivatization and LC-TOF/MS conditions are described in the Methods section. (**b**) Changes in brain/perfusate ratio of [^13^C_2_,^15^N]Gly-Sar with perfusion time. FITC-albumin was used as negative control (BBB non-transportable compound). *In situ* mouse perfusion experiments were performed using [^13^C_2_,^15^N]Gly-Sar (200 μM) or FITC-albumin (100 µg/mL) at a perfusion flow rate of 2.0 mL/min, as described in the Methods section (n = 4 for [^13^C_2_,^15^N]Gly-Sar and n = 3 for FITC-albumin). Calculated *K*_*i*_ values are represented in the figure. Results are expressed as the mean ± s.e.m. Significant difference between brain/perfusate ratios of FITC-albumin and peptide groups over time was analyzed by two-way ANOVA, followed by Bonferroni *post hoc* test. ***P < 0.001 as compared to FITC-albumin.

In order to elucidate the entry of dipeptides into the brain, Gly-Sar, a model substrate for SLC15 family of peptide transporters^16^, was selected to validate the present *in situ* mouse brain perfusion experiments. The transport of Gly-Sar across mouse BBB was determined in brain homogenates by using 2,4,6-trinitrobenzensulfonate (TNBS) aided-liquid chromatography-time-of-flight (LC-TOF)/MS technique described previousely^17^, wherein the target peptide was derivatized with TNBS to form a trinitrophenyl (TNP)-peptide. Since Gly-Sar ([M + H]^+^: 358.0630 *m*/*z*) has the same molecular mass as endogenous Gln, a stable isotope labeled [^13^C_2_,^15^N]Gly-Sar ([M + H]^+^: 361.0630 *m*/*z*), which was used for selective MS detection of perfused Gly-Sar. As shown in Fig. 1a, a time-dependent increase in the uptake of [^13^C_2_,^15^N]Gly-Sar was clearly observed during the indicated perfusion time of 0 to 2 min. Brain/perfusate ratio of [^13^C_2_,^15^N]Gly-Sar and the influx rate constant (*K*_*i*_ value: 7.60 ± 1.29 µL/g·min) were significantly (*P* < 0.01) higher than those of fluorescein isothiocyanate conjugated (FITC)-albumin, a compound that cannot cross the BBB^18^ (Fig. 1b). This observation strongly suggested that a dipeptidic compound, Gly-Sar, may transport across the BBB in the blood-to-brain direction. No increase in the brain/perfusate ratio of FITC-albumin during the co-perfusion with Gly-Sar (Fig. S1) clearly indicated that Gly-Sar transport did not cause any BBB disruption (Fig. 1a).

**Figure 2:**
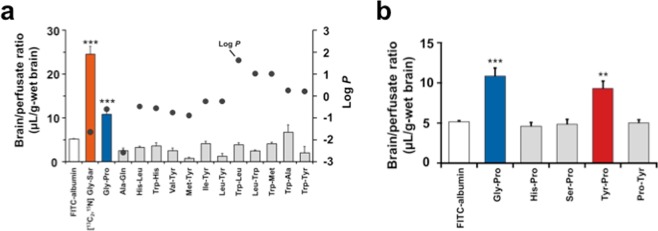
Transport efficiency of dipeptides across BBB. (**a**) Brain/perfusate ratio of dipeptides. Thirteen dipeptides (Gly-Pro, Ala-Gln, His-Leu, Trp-His, Val-Tyr, Met-Tyr, Ile-Tyr, Leu-Tyr, Trp-Leu, Leu-Trp, Trp-Met, Trp-Ala, and Trp-Tyr; 200 μM each) were used for 2.0-min brain perfusion experiments. FITC-albumin (100 µg/mL) was used as the negative control, while [^13^C_2_,^15^N]Gly-Sar (200 μM) was used as positive control. *In situ* mouse perfusion experiments were performed at a perfusion flow rate of 2.0 mL/min, as described in the Methods section (n = 4 for dipeptides; n = 3 for FITC-albumin). Hydrophobicity (log *P* value) of each dipeptide was obtained by a SciFinder Substance Identifier software (https://scifinder.cas.org/scifinder/view/scifinder/scifinderExplore.jsf). Results are expressed as the mean ± s.e.m. ***P < 0.001 as compared to FITC-albumin using unpaired two-tailed Student’s *t*-test. (**b**) Brain/perfusate ratio of dipeptides with Pro at the C-terminus. Five dipeptides (Gly-Pro, His-Pro, Ser-Pro, Tyr-Pro, and Pro-Tyr; 200 μM each) were used for 2.0-min brain perfusion experiments. The *in situ* mouse perfusion experiments were identical to the above experiment (**a**). (n = 4 for dipeptides; n = 3 for FITC-albumin). Results are expressed as the mean ± s.e.m. **P < 0.01 and ***P < 0.001 as compared to FITC-albumin using Dunnett’s *t*-test.

**Figure 3:**
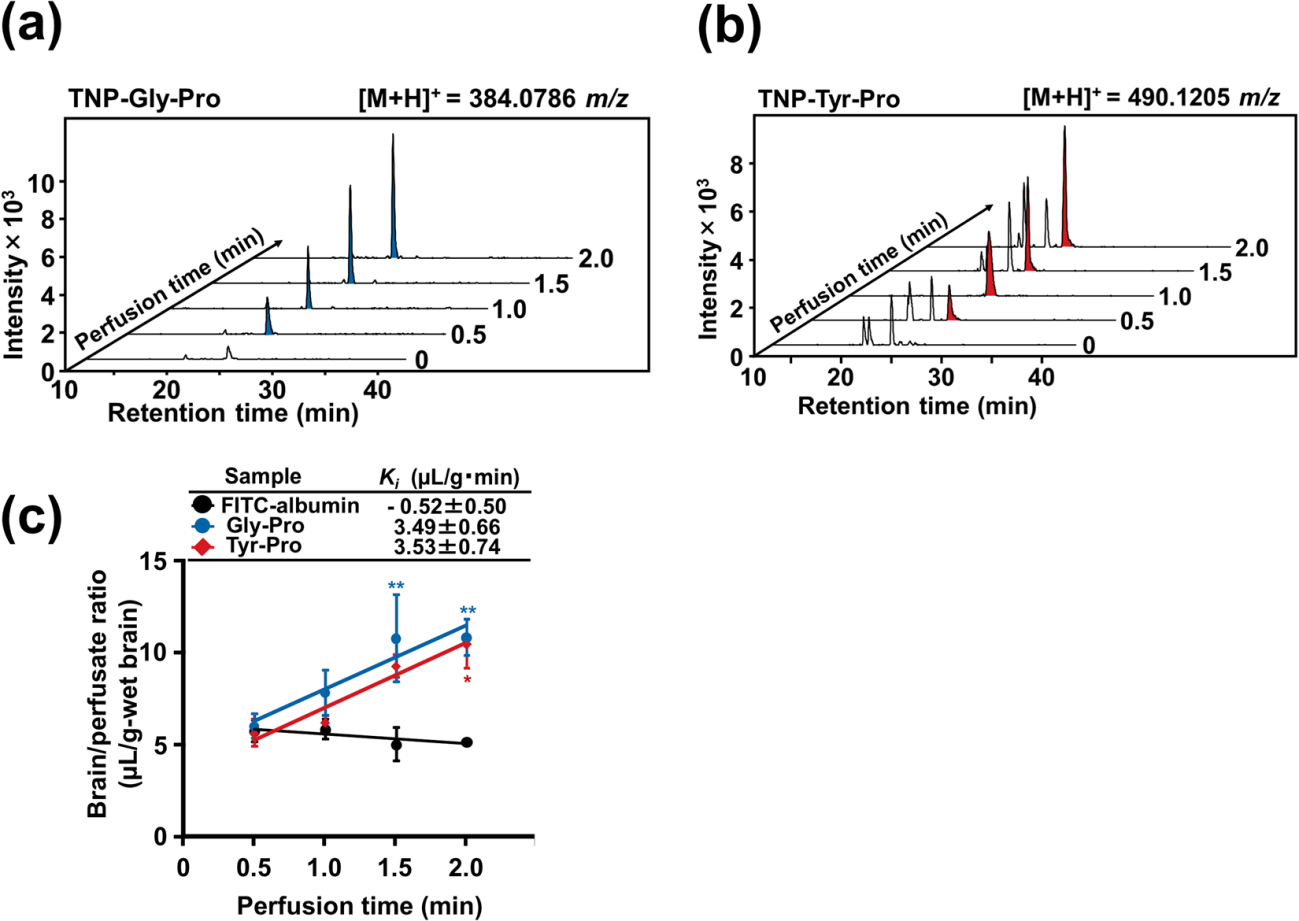
Transport of Gly-Pro and Tyr-Pro across BBB. Stacked extracted ion chromatograms of TNP-Gly-Pro ([M + H]^+^, 384.0786 *m*/*z*) (**a**) and TNP-Tyr-Pro ([M + H]^+^, 490.1205 *m*/*z*) (**b**) of perfused brain at interval times of 0, 0.5, 1.0, 1.5, and 2.0 min, respectively, as examined by LC-TOF/MS. TNBS derivatization and LC-TOF/MS conditions are described in the Methods section. (**c**) Changes in brain/perfusate ratio of Gly-Pro and Tyr-Pro with perfusion time. FITC-albumin was used as the negative control. Mouse perfusion conditions were the same as described in Fig. 1. Results are expressed as the mean ± s.e.m. Significant difference between brain/perfusate ratios of FITC-albumin and peptide groups over time was analyzed by two-way ANOVA, followed by Bonferroni *post hoc* test. **P < 0.01 as compared to FITC-albumin.

After confirming the intact transport of dipeptidic Gly-Sar across the mouse BBB, further *in situ* mouse brain perfusion experiments for BBB transportable peptides were performed on the basis of the dipeptide skeleton. Gly-Pro, Ala-Gln, His-Leu, Trp-His, Val-Tyr, Met-Tyr, Ile-Tyr, and Leu-Tyr were selected, since they have been reported to be transported in an intact form through the intestinal membrane via carrier-mediated routes^3,4,19^. Additionally, by considering that the hydrophobicity may assist the transcellular diffusive transport^20,21^, Trp-Leu, Leu-Trp, Trp-Met, Trp-Ala, and Trp-Tyr with log *P* values of 1.62, 1.02, 1.00, 0.25, and 0.21, respectively, were also assayed. After 2.0-min perfusion of all the 13 peptides, only Gly-Pro showed a significant (*P* < 0.001) uptake with a brain/perfusate ratio of 10.9 ± 0.1 μL/g-brain, compared to FITC-albumin (Fig. 2a). This indicates that hydrophobicity is not a key factor in determining the intact BBB transport of dipeptides within the present experimental conditions. The observation that a dipeptide, Gly-Pro, as well as Gly-Sar can be transported in their intact forms across the mouse BBB, led us to speculate that dipeptides with an imino bond could be possible BBB-transportable candidates. Hence, four dipeptides containing Pro at the C-terminal including Gly-Pro, His-Pro, Ser-Pro, and Tyr-Pro, were targeted for further screening of BBB-transportable dipeptides. As shown in Fig. 2b, it was clear that Tyr-Pro was a BBB-transportable dipeptide (brain/perfusate ratio: 10.5 ± 1.3 μL/g-brain), with efficiency comparable to that of Gly-Pro (10.9 ± 0.1 μL/g-brain). No increase in brain uptake for Pro-Tyr (Fig. 2b) strongly suggested the importance of positioning of Pro at the C-terminus of dipeptide skeleton. *In situ* mouse brain perfusion experiments revealed for the first time that two dipeptides, Gly-Pro and Tyr-Pro, with significant transport capacity (*K*_*i*_ value) of 3.49 ± 0.66 and 3.53 ± 0.74 µL/g·min, respectively, are capable of crossing the controlled BBB system (Fig. 3) (c.f., *K*_*i*_ of Gly-Sar: 7.60 ± 1.29 µL/g·min, Fig. 1).

### BBB transport of Gly-Sar and Tyr-Pro into brain parenchyma

**Figure 4:**
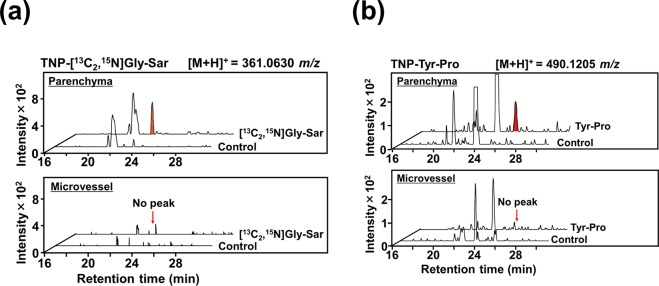
LC-TOF/MS detection of Gly-Sar and Tyr-Pro in the parenchyma of brain. Extracted ion chromatograms of TNP-[^13^C_2_,^15^N]Gly-Sar ([M + H]^+^, 361.0630 *m*/*z*) (**a**) and TNP-Tyr-Pro ([M + H]^+^, 490.1205 *m*/*z*) (**b**) in the parenchyma and microvessel fractions of mouse brain by LC-TOF/MS. Each fraction was prepared from 2.0-min peptide (200 μM) perfused brain, as described in the Methods section. Perfusion of the buffer in the absence of dipeptide at the same perfusion conditions was denoted as control in the chromatograms.

To validate the intact BBB transport of dipeptides and accumulation of perfused [^13^C_2_,^15^N]Gly-Sar and Tyr-Pro after 2.0-min perfusion, and also to rule out the non-specific binding of the dipeptides with the capillaries in the brain, the mouse brain parenchyma was collected by brain capillary depletion technique using dextran density centrifugation^22^. As shown in Fig. 4, both [^13^C_2_,^15^N]Gly-Sar and Tyr-Pro were enriched in the mouse brain parenchyma, as detected by the TNBS-LC-TOF/MS, but were undetectable in the brain microvessel fraction, suggesting that both Gly-Sar and Tyr-Pro were transported across the BBB in intact form into the mouse brain parenchyma.

**Figure 5:**
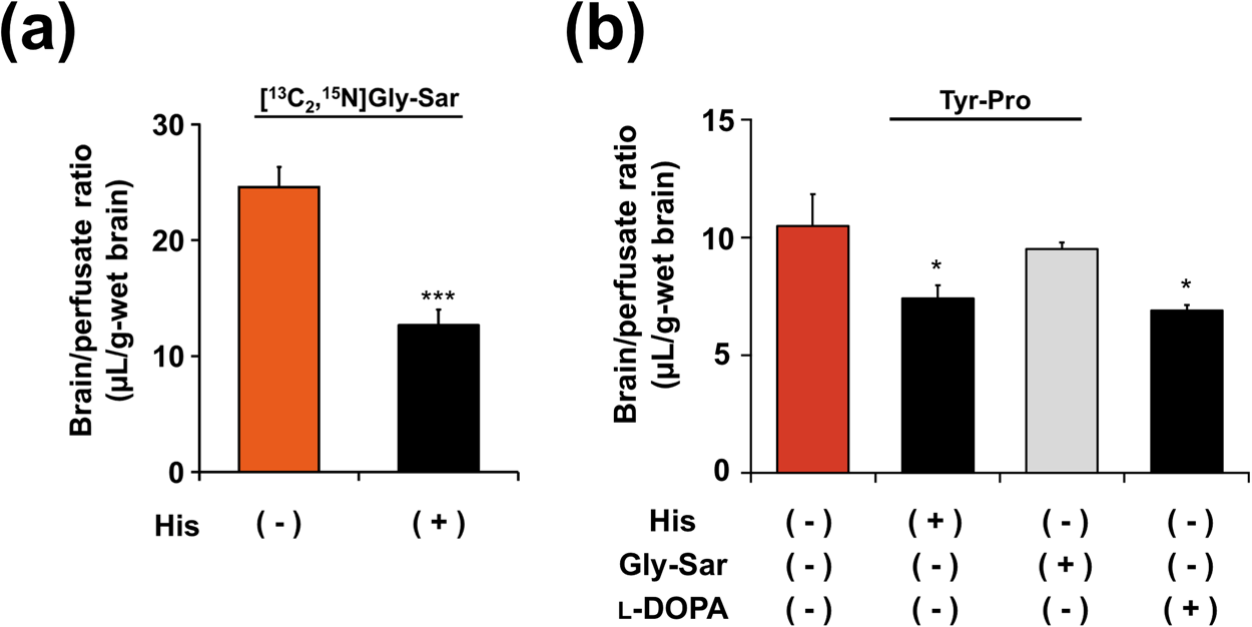
Effect of histidine on brain/perfusion ratio of Gly-Sar (**a**) and effect of histidine, Gly-Sar, or l-DOPA on brain/perfusion ratio of Tyr-Pro (**b**) in *in situ* brain perfusion experiments. *In situ* mouse perfusion experiments for 2.0 min were performed by [^13^C_2_,^15^N]Gly-Sar (200 μM) or Tyr-Pro (200 μM) in the presence or absence of inhibitor (5 mM) at a perfusion flow rate of 2.0 mL/min, as described in the Methods section (n = 4). Results are expressed as the mean ± s.e.m. ***P < 0.001 and *P < 0.05 using unpaired two-tailed Student’s *t*-test for Gly-Sar and Dunnett’s *t*-test for Tyr-Pro.

The possible BBB transport route of both dipeptides was examined by co-perfusion with His [as reported substrate of brain PHT1^5^ and/or l-type amino acid transporter 1 (LAT1)^23^] or Gly-Sar (as PHT1 substrate)^12^. As shown in Fig. 5a,b, His significantly reduced the brain/perfusate ratios of [^13^C_2_,^15^N]Gly-Sar and Tyr-Pro, suggesting that both dipeptides might be transported across the BBB via a common carrier, PHT1. However, no altered uptake of Tyr-Pro by Gly-Sar and reduced uptake by l-DOPA were obtained (Fig. 5b). Moreover, the brain/perfusate ratio of 2.0 min perfusion of Gly-Sar at 5 mM (18.1 ± 0.5 µL/g-brain) in the present experimental condition was significantly lower than that at 200 µM (24.5 ± 1.8 µL/g-brain), which indicated the concentration of Gly-Sar used in the present co-perfusion experiments was enough to be saturated for BBB transport of Gly-Sar. These results strongly suggested that an LAT1 (or other) transport route(s) cannot be ruled out for the BBB transport of Tyr-Pro. BBB transport route(s) of the dipeptides are now in investigation using brain capillary endothelial cells.

### Location of Tyr-Pro in mouse brain by MALDI-MS imaging

**Figure 6:**
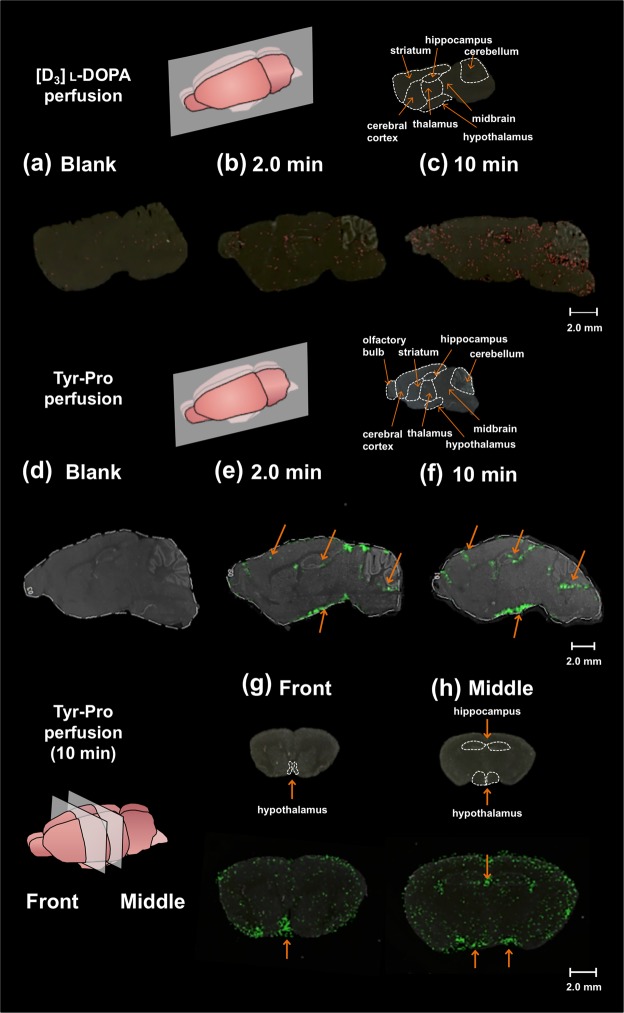
Visualization of [D_3_]l-DOPA and Tyr-Pro in perfused mouse brain by MALDI-MS imaging. MALDI-MS images of [D_3_]l-DOPA ([M–H]^−^, 199.1 *m*/*z*) were obtained using the sagittal tissue sections of 2.0-min or 10-min [D_3_]l-DOPA (200 μM) perfused mouse brain ((**a**) blank; (**b**) 2.0 min; (**c**) 10 min). MALDI-MS/MS images of Tyr-Pro ([M + H]^+^, 279.1 > 116.0 *m*/*z*) were obtained from sagittal tissue sections of 2.0 min or 10 min Tyr-Pro (200 μM) perfused mouse brain ((**d**) blank; (**e**) 2.0 min; (**f**) 10 min). MALDI-MS/MS images of Tyr-Pro from coronal sections were also obtained from 10 min perfused mouse brain ((**g**) front; (**h**) middle). The conditions of MALDI-TOF/MS imaging analysis are described in the Methods section. Data are shown using a color gradient, normalized against the total ion count. Scale bar, 2 mm; spatial resolution = 90 µm.

Based on the evidence that Tyr-Pro is transported across the BBB into brain parenchyma (Fig. 4), MALDI-MS imaging visualization analyses were performed to determine the regions of peptide accumulation. The brains were monitored after 2.0-min and 10-min perfusions of Tyr-Pro, respectively. l-DOPA that can be transported across the BBB^23^ was selected as the positive control to validate the visualization analysis. [D_3_]l-DOPA was used for perfusion to distinguish it from endogenous l-DOPA during MS detection. As shown in Fig. 6a–c, the distribution of [D_3_]l-DOPA could be effectively visualized in the mouse brain tissues upon increasing the perfusion time up to 10 min. MALDI-MS/MS imaging indicated that Tyr-Pro (target mass of fragment Pro: [M + H]^+^, 279.1 > 116.0 *m*/*z*) was transported across the BBB and accumulates in the hippocampus, hypothalamus, striatum, cerebral cortex, and cerebellum of the mouse brain (Fig. 6d–f: sagittal slice, g and h: coronal slice).

## Discussion

The brain maintains its characteristic homeostasis by showing selective uptake of nutrients, and also by excluding many compounds and metabolites, including peptides by the BBB system between circulating blood and the brain^8^. Although previous reports suggest that some di-/tripeptides can be absorbed intact across intestinal membrane^3,4^, their transport beyond the BBB still needs to be studied in detail. In contrast, recent studies report the beneficial effects of peptide intake in improving memory and cognitive functions^13,14,24^.

The uptake of dipeptidic Gly-Sar in brain through PHT1 in adults or PepT2 pathway in neonatal rodents by mouse brain perfusion experiments was the first *ex vivo* report of peptide transport across the BBB^12^. This observation strongly led us to investigate if dipeptides can be transported beyond the BBB *in vivo*. In the present study, using *in situ* mouse perfusion experiments for up to 2.0 min, we demonstrate for the first time that dipeptides, Gly-Pro and Tyr-Pro, along with Gly-Sar, are transported across the BBB to be accumulated in their intact form in the brain parenchyma by using LC-TOF/MS (Fig. 4). Although no compatible experiments have been performed, the pharmacokinetics data obtained using the *in situ* perfusion experiments showed comparable or high *K*_*i*_ values of 7.60 ± 1.29, 3.49 ± 0.66, and 3.53 ± 0.74 µL/g·min for Gly-Sar, Gly-Pro, and Tyr-Pro, respectively, when compared to the reported *K*_*i*_ values of 3 µL/g·min for neuropeptide Y (36 amino acids)^25^, 0.84 µL/g·min for growth hormone (191 amino acids)^26^, and 0.57 µL/g·min for peptide hormone ghrelin (28 amino acids)^27^. The comparative brain/perfusate ratio of 19.3 ± 5.9 µL/g-brain for a reported BBB-transportable l-DOPA^28^ (data were not shown in Fig. 2) with those of Gly-Sar (24.5 ± 1.8 µL/g-brain) and Tyr-Pro (10.5 ± 1.3 µL/g-brain) (Fig. 2) also suggested acceptable BBB transportability of both dipeptides. When the BBB is disrupted by brain injury, the transport across the BBB is altered significantly; for example, a marked increase in the uptake of BBB non-transportable FITC-albumin was documented upon BBB disruption^18^. Our study provides the first evidence of an intact dipeptide transport beyond the BBB (Fig. 2) without any BBB disruption. Exclusion of FITC-albumin^18^ from the 5.0 min co-perfusion with Gly-Sar (Fig. S1) clearly indicates that *in situ* mouse perfusions did not cause any BBB disruption, and also confirmed the transport of the dipeptide across the mouse BBB.

Since hydrophobicity of the dipeptides did not facilitate their uptake in brain (Fig. 2), we concluded that the transcellular diffusive pathways, through which hydrophobic compounds such as CPP^29,30^, delta sleep-inducing peptide (DSIP)^20^, and an anxiolytic diazepam^31^ were transported, might not be involved in the transport of these dipeptides across the BBB. Hitherto, no influx carrier mechanism for peptide transport into brain parenchyma across the BBB has been elucidated. However, it has been reported that PepT2, expressed in the apical membrane of choroid plexus epithelial cells and plasma membrane of neural cells is involved in the removal of peptidic substrates from the CSF^32^. In this study, a significant reduction in the uptake of Gly-Sar by co-perfusion of His, a favorable PHT1 substrate^5^ (Fig. 5a), suggested that PHT1 might be a possible carrier for Gly-Sar. This is consistent with the observation that the uptake of Gly-Sar is via PHT1 in brain tissue slices^12^. On the other hand, the brain uptake of Tyr-Pro was not altered by co-perfusion with Gly-Sar (Fig. 5b), suggesting that Tyr-Pro may get transported across the BBB via an independent transport system. In co-perfusion experiments of Tyr-Pro with His or l-DOPA, both of which are recognized by l-type amino acid transporter 1 (LAT1)^23^, we observed that the brain uptake of Tyr-Pro was affected by both His and l-DOPA (Fig. 5b), suggesting that an LAT1 (or other) transport route(s) might be involved in the BBB transport of Tyr-Pro. However, further experiments with e.g., receptor antagonists and LAT1 knockdown in brain capillary endothelial cells are required to clarify the BBB transport route(s) for these dipeptides.

We also show that phytic acid-aided MALDI-MS imaging technique^15^ can be used to visualize the histological distribution of Tyr-Pro in the mouse brain. As shown in Fig. 6a–c, we were able to visualize [D_3_]l-DOPA throughout the brain after 2.0-min and 10-min perfusions, respectively. This is consistent with the distribution of [^18^F]fluorodopa in human brain by positron emission tomographic (PET) study^33^, supporting the use of the present MALDI-MS imaging technique to elucidate the distribution of BBB-transportable dipeptides in the mouse brain. In this study, we demonstrate the localization of perfused Tyr-Pro for 2.0 min and 10 min, respectively, in the hippocampus, hypothalamus, cerebral cortex, and cerebellum of the mouse brain (Fig. 6d–f: sagittal slices, g and h: coronal slices). Thus far, there have been no reports on the distribution of dipeptides in brain, or on the beneficial effects of the same on the brain, except for the evidential improvement of delayed memory score in patients with mild cognitive impairment by the intake of protein hydrolysate^13^. Although Tyr-Pro, which was present in soybean hydrolysate at 0.47 mg/g-hydrolysate (Fig. S2), showed *in vitro* angiotensin I-converting enzyme inhibition^34^ and antioxidant effects^35^, there is no study supporting its *in vivo* benefits. Therefore, the current observation of the accumulation of *in vivo* Tyr-Pro in the regions of the brain involved in memory consolidation and spatial memory (hippocampus^36^), food intake control (hypothalamus), and cognition (cerebrum and cerebellum^37^) suggests that the localization of dipeptides could have a potential physiological role in the brain. It will, therefore, be interesting to examine the physiological effects and bioavailability of orally administered Tyr-Pro in animal models.

In conclusion, this study provides evidence for the transport of intact Gly-Pro and Tyr-Pro, along with dipeptidic Gly-Sar, across the BBB into the mouse brain parenchyma. Moreover, we also show the selective accumulation of Tyr-Pro in the hippocampus, hypothalamus, striatum, cerebral cortex, and cerebellum of mouse brain by phytic acid-aided MALDI-MS imaging technique. Although further studies are needed to point out the physiological roles of the BBB-transportable dipeptides in the brain, we establish dipeptides as molecules capable of penetrating the BBB.

## Methods

### Chemicals and reagents

Gly-Sar, aprotinin, and FITC-labeled albumin were obtained from Sigma-Aldrich (St. Louis, MO, USA). [^13^C_2_,^15^N]-labeled Gly-Sar and [^13^C_5_,^15^N]-labeled Tyr-Pro were synthesized by Scrum Co. (Tokyo, Japan). [D_3_]-labeled l-DOPA was obtained from Cambridge Isotope Laboratories Inc. (Tewksbury, MA, USA). Chymostatin was purchased from Peptide Institute Inc. (Osaka, Japan). Dipeptides (Gly-Sar, Gly-Pro, Ala-Gln, His-Leu, Trp-His, Val-Tyr, Met-Tyr, Ile-Tyr, Leu-Tyr, Trp-Leu, Leu-Trp, Trp-Met, Trp-Ala, Trp-Tyr, His-Pro, Ser-Pro, Tyr-Pro, and Pro-Tyr) were purchased from Kokusan Chemical Co. (Osaka, Japan). TNBS was purchased from Nacalai Tesque Co. (Kyoto, Japan). 1,5-Diaminonaphthalene (1,5-DAN) was procured from Tokyo Chemical Industry Co. (Tokyo, Japan). Distilled water, methanol (MeOH), acetonitrile (ACN), and formic acid (FA); each reagent of LC-MS grade, were purchased from Merck Co. (Darmstadt, Germany). All other reagents used, were of analytical grade and were used without further purification.

### Animals

Seven to nine-week old male ICR mice with 30–40 g body weight (Jcl:ICR, CLEA Japan, Tokyo, Japan) were used in this study. All mice were housed for 1 week under controlled temperature at 21 ± 1 °C, humidity at 55 ± 5%, and light scheduled for a twelve-hour period from 8:00 am to 8:00 pm. The mice were fed the laboratory diet (CE-2, CLEA Japan) and water *ad libitum*. All animal experiments in this study were handled in accordance with the Proper Conduct of Animal Experiments and Related Activities in Academic Research Institutions under the jurisdiction of the Ministry of Education, Culture, Sports, Science, and Technology in Japan. The Ethics Committee on Animal Experiments at Fukuoka University approved all experimental protocols (permit number: 1606938 and 1702014).

### *In situ* transcardiac mouse brain perfusion experiments

*In situ* mouse brain perfusion experiments were conducted as previously described^38^ with slight modifications. Briefly after mice were anesthetized with 40% urethane (Sigma-Aldrich, St. Louis, MO, USA), the descending thoracic aorta was ligated, and at the start of perfusion, left jugular was sectioned. Freshly prepared perfusion fluid [120 mM NaCl, 4 mM KCl, 2.5 mM CaCl_2_, 25 mM NaHCO_3_, 1.2 mM KH_2_PO_4_, 1.8 mM anhydrous MgCl_2_, 5.5 mM d-glucose, and 1% BSA], containing FITC-albumin (200 µM), peptides (200 µM) or (D_3_)l-DOPA (200 µM)] was infused in the left ventricle of the heart by inserting a 26-gauge butterfly needle at a rate of 2.0 mL/min for 0–10 min (n = 3/FITC-albumin and n = 4/peptide group). For co-perfusion of peptides with Gly-Sar (5 mM), His (5 mM) or l-DOPA (5 mM), freshly prepared perfusion fluid containing each compound was infused as mentioned above for 2.0 min (n = 4 for each group). After perfusion, whole brain was removed from the mice by decapitation and weighed. Brain samples for quantitative analysis by LC-TOF/MS were immediately frozen in liquid nitrogen, whereas the samples for MALDI-MS imaging analysis were immediately frozen using powdered dry ice to avoid any degradation of tissue shape. All brain samples were stored at −80 °C until analysis was performed.

### Preparation of brain parenchyma and microvessel fractions

The brain parenchyma and microvessel fractions were prepared as described by Triguero *et al*.^22^. Brain was obtained by decapitation after 2.0-min dipeptide perfusion, and arachnoid membranes were peeled away. The isolated brain was minced using a glass homogenizer in 0.5 mL physiological buffer used for preparation of the perfusion fluid, followed by further homogenization after the addition of 1.0 mL of 26.5% dextran at 4 °C. The homogenate thus obtained was centrifuged at 5,400 × *g* for 15 min at 4 °C. The supernatant representing the parenchyma fraction, and the pellet representing the microvessel fraction were carefully collected (Fig. S3). One milliliter of the physiological buffer was then added to the supernatant and centrifuged at 5,400 × *g* for 15 min at 4 °C to obtain the brain parenchyma. The absence of parenchyma debris contamination in the brain microvessel fraction was confirmed by microscopy (Fig. S3). For the pellet, 1.5 mL of 17.7% dextran was added, followed by centrifugation at 5,400 × *g* for 15 min at 4 °C to obtain the microvessel fraction. Both parenchyma and microvessel fractions were frozen and stored at −80 °C.

### Quantification of perfused dipeptides in brain by liquid chromatography-mass spectrometry

Frozen whole brain, brain parenchyma and microvessel fractions after the perfusion of dipeptides were lyophilized and mashed with a BioMasher II (Nippi. Inc., Tokyo, Japan). An aliquot (10 mg) of the obtained homogenous powder was dissolved in 1.0 mL of homogenized buffer (0.1% NaCl and 0.1% FA) containing [^13^C_5_,^15^N]Tyr-Pro (60 pmol/mL) as the internal standard (IS) and protease inhibitors (0.5 mg/mL ethylenediaminetetraacetic acid disodium salt (EDTA-2Na), 0.1 mg/mL aprotinin, and 0.1 mg/mL chymostatin). The sample solution was sonicated using a SONIFIRE 250 (Branson Ultrasonics, Emerson Japan Co., Kanagawa, Japan) with an output control of 3 for 10 s × 3 times at 4 °C, following the homogenization with a Polytron homogenizer (KINEMATICA AG, Luzern, Switzerland) with 20,000 rpm for 30 s × 3 times at 4 °C. After the centrifugation of the homogenate at 14,000 × *g* for 15 min at 4 °C, the supernatant obtained was subjected to ultrafiltration using an Amicon Ultra 0.5-mL-3K centrifugal filter (Millipore, Carrigtwohill, Ireland) at 14,000 × *g* for 30 min at 4 °C. The collected filtrate was evaporated till it dried. The dry filtrate was then subjected to a TNBS derivatization^17^ to obtain TNP-derivatives. 50 µL of TNBS solution (150 mM, pH 10) was added to the filtrate, and incubated for 30 min at 30 °C. After the addition of 50 µL of 0.2% FA solution to stop the TNBS reaction, an aliquot (20 µL) of the solution was subjected to LC-TOF/MS analysis.

LC-TOF/MS analysis was performed as follows: LC separation was performed using an Agilent 1200 series system (Agilent, Waldbronn, Germany) on a Biosuite Peptide column (2.1 × 150 mm, 3 µm, Waters, Milford, MA, USA) at 40 °C with a linear gradient elution of MeOH (0–100% over 20 min) containing 0.1% FA at a flow rate of 0.25 mL/min. Electrospray ionization (ESI)-TOF/MS analysis was carried out using a microTOF II equipment (Bruker Daltonics, Bremen, Germany) in positive mode. The ESI conditions were as follows: drying gas (N_2_), 8.0 L/min; drying temperature, 200 °C; nebulizing gas (N_2_), 1.6 bar; capillary voltage, 4,500 V; and mass range, 100–1,000 *m*/*z*. All data acquisition and analyses were performed by using Bruker Data Analysis 3.2 software. A calibration solution containing 10 mM sodium formate in 50% ACN was injected at the beginning of each run, and all spectra were internally calibrated.

Typical calibration graphs obtained with the aforementioned MS conditions were used to determine the amount of dipeptides in dry brain weight (g) as follows: [^13^C_2_,^15^N]Gly-Sar, *y* = 2,086*x* + 16 (*r* = 0.994); Gly-Pro, *y* = 1,656*x* + 45 (*r* = 0.987); Ala-Gln, *y* = 1,675*x* + 13 (*r* = 0.926); His-Leu, *y* = 10,263*x* − 39 (*r* = 0.974); Trp-His, *y* = 1,304*x* − 1 (*r* = 0.999); Val-Tyr, *y* = 1,453*x* + 9 (*r* = 0.996); Met-Tyr, *y* = 1,332*x* + 9 (*r* = 0.994); Ile-Tyr, *y* = 6,822*x* + 53 (*r* = 0.979); Leu-Tyr, *y* = 4,313*x* + 169 (*r* = 0.994); Trp-Leu, *y* = 3,258*x* − 11 (*r* = 0.999); Leu-Trp, *y* = 2381*x* + 46 (*r* = 0.959), Trp-Met, *y* = 1,436*x* − 13 (*r* = 0.978); Trp-Ala, *y* = 4,732*x* + 21 (*r* = 0.997); Trp-Tyr, *y* = 2,667*x* − 18 (*r* = 0.990); His-Pro, *y* = 1,843*x* − 10 (*r* = 0.979); Ser-Pro, *y* = 2,135*x* + 46 (*r* = 0.994); Tyr-Pro, *y* = 2,058*x* − 3 (*r* = 0.999), and Pro-Tyr, *y* = 1,932*x* − 1 (*r* = 0.999) [where *y* is the peak area ratio (observed peak area of the target to that of IS) and *x* is the peptide concentration between 0–18 nmol/g-dry brain]. Brain/perfusate ratio of the perfused peptide was calculated by the peptide concentrations in wet brain (converted from dry brain weight) as follows: brain/perfusate ratio (µL/g wet brain) = concentration of perfused peptide in wet brain (nmol/g wet brain)/concentration of peptide in perfusate (nmol/µL). *K*_i_ value (µL/g·min) was obtained by calculating the slope of brain/perfusate ratio against perfusion time. The kinetics experiments were performed 4 times each, at the individual time intervals of 0.5, 1.0, 1.5, and 2.0 min, respectively.

### MALDI-MS imaging analysis

Distribution of the perfused Tyr-Pro and [D_3_]l-DOPA in the mouse brain was analyzed using the proposed phytic acid-aided MALDI-MS imaging technique^15^. A frozen whole brain after peptide perfusion experiments was sliced into 12-μm-thick sections at both sagittal and coronal faces using a CM1850 Leica Cryomicrotome (Leica, Wetzler, Germany). Each section was thaw-mounted on an indium-tin oxide (ITO)-coated conductive glass slide (Bruker Daltonics) and dried under the N_2_ gas flow. DHB was used as MALDI matrix, for the detection of Tyr-Pro in positive MS mode. For matrix preparation, DHB (50 mg/mL) was dissolved in MeOH/water (1:1, v/v) containing 50 mM phytic acid^15^ and 250 mM (NH_4_)_2_SO_4_, while 1,5-DAN (10 mg/mL) in 70% ACN was used for the detection of [D_3_]l-DOPA in negative MS mode. Each matrix was sprayed with an ImagePrep automatic matrix sprayer (Bruker Daltonics) over the ITO-glass slide. MALDI-MS imaging analysis for brain tissue-mounted ITO-glass slide was performed using an ultrafleXtreme mass spectrometer equipped with a smartbeam II Laser (Bruker Daltonics) in reflector and LIFT modes. MS/MS data were acquired with monoisotopic isolation of 279.1 > 116.0 *m*/*z* for Tyr-Pro, while MS data were acquired with 199.1 *m*/*z* for [D_3_]l-DOPA. MS parameters were as follows: ion source voltage 20.00 kV; reflector voltage, 20.8 kV; lens voltage, 6.50 kV; number of shots, 100 shots/spot; laser frequency, 1000 Hz; laser focus, medium. MS imaging analysis was performed with a spatial resolution of 90 μm. The image data were constructed for visualization with mass filters of ±0.01 *m*/*z* for [D_3_]l-DOPA in MS mode, and ±0.3 *m*/*z* for Tyr-Pro in MS/MS mode by Bruker flexImaging software (ver. 4.1).

### Statistical analyses

Results are expressed as the mean ± standard error of the mean (s.e.m.). Statistical evaluation between two groups was performed by using an unpaired two-tailed Student’s *t*-test. A one-way analysis of variance (ANOVA) was performed to analyze the difference among more than three groups, followed by Dunnett’s *t*-test for post hoc analysis. A two-way ANOVA was performed to analyze the difference between brain/perfusate ratios of FITC-albumin and peptide groups over time, followed by Bonferroni *post hoc* test. A *P* value of <0.05 was considered significant. All statistical analyses were carried out using GraphPad Prism 5 software (GraphPad Software, La Jolla, CA, USA).

## Supplementary information

Supplemental figures

## Data Availability

The data supporting the findings reported herein are available on request, from the corresponding author.
