# Supplementary material for: Brain-transportable dipeptides across the blood-brain barrier in mice
Source: Sci Rep. 2019 Apr 8;9:5769. doi: 10.1038/s41598-019-42099-9 (PMC6453885; doi:10.1038/s41598-019-42099-9)
Supplement: Supplementary file 1 — Supplemental figures [file 41598_2019_42099_MOESM1_ESM.docx]

**Supplementary information**

**Brain-transportable dipeptides across the blood-brain barrier in mice**

**Authors:** Mitsuru Tanaka^1#^, Shinya Dohgu^2#^, Genki Komabayashi^1^, Hayato Kiyohara^1^, Fuyuko Takata^2^, Yasufumi Kataoka^2^, Takashi Nirasawa^3^, Motohiro Maebuchi^4^, Toshiro Matsui^1^*

**Affiliations:**

^1^Department of Bioscience and Biotechnology, Faculty of Agriculture, Graduate School of Kyushu University, 744 Motooka, Nishi-ku, Fukuoka 819-0395, Japan

^2^Department of Pharmaceutical Care and Health Sciences, Faculty of Pharmaceutical Sciences, Fukuoka University, 8-19-1 Nanakuma, Jonan-ku, Fukuoka 814-0180, Japan

^3^Bruker Japan K.K., 3-9, Moriya-cho, Kanagawa-ku, Yokohama, Kanagawa 221-0022, Japan

^4^FUJI OIL CO., Ltd., Research and Development Division, 1 Sumiyoshi-cho, Izumisano, Osaka 598-8540, Japan

^*^**Correspondence**: Toshiro Matsui, PhD, Department of Bioscience and Biotechnology, Faculty of Agriculture, Graduate School of Kyushu University, 744 Motooka, Nishi-ku, Fukuoka 819-0395, Japan

**E-mail:** tmatsui@agr.kyushu-u.ac.jp

**Fax:** +81-92-802-4752

**
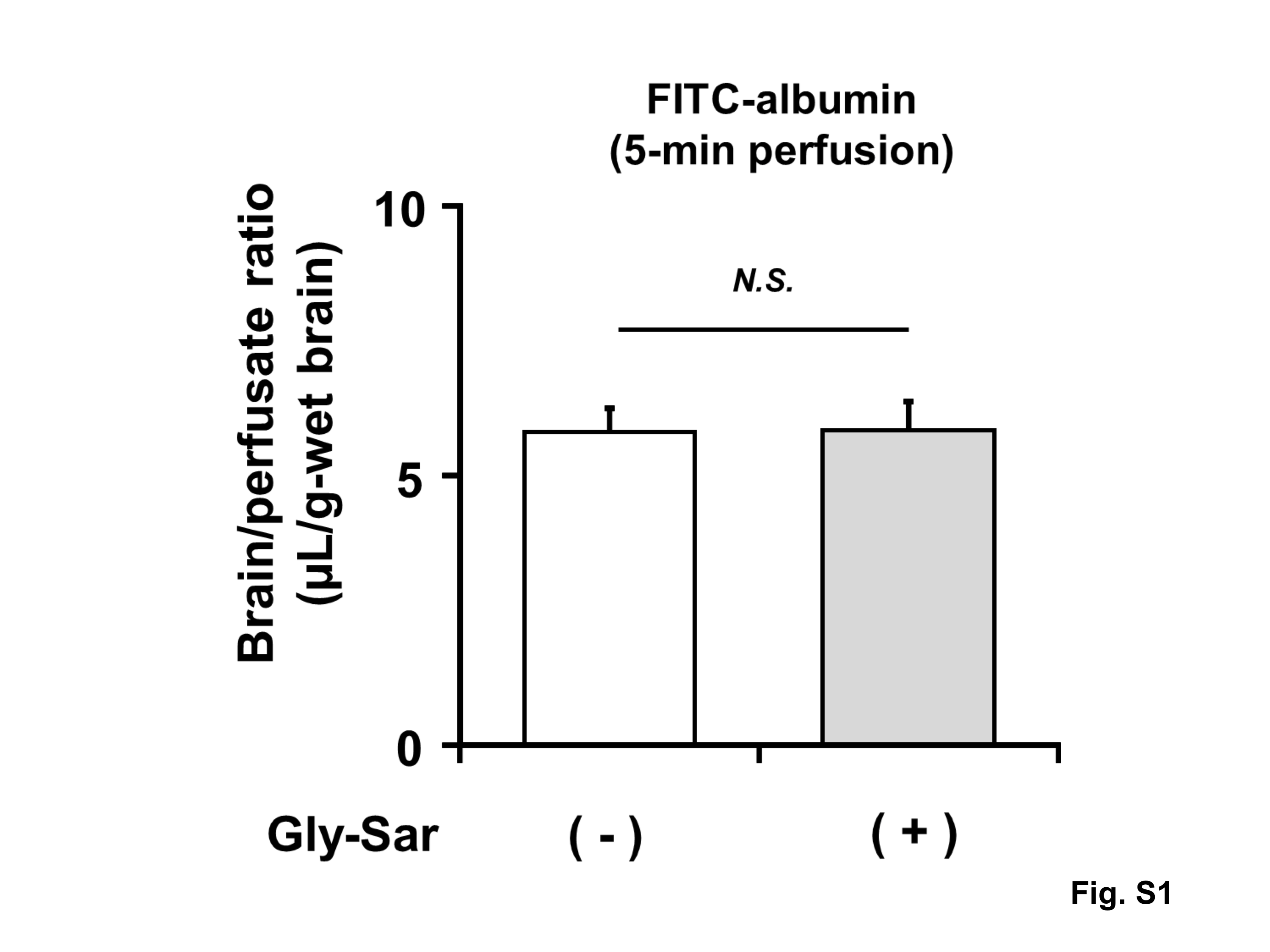
**

**Fig. S1. Effect of Gly-Sar on brain/perfusate ratio of FITC-albumin in *in situ* ICR mouse brain perfusion experiments.** Brain perfusion experiments were performed using FITC-albumin (100 µg/mL) in the presence or absence of Gly-Sar (200 μM) at a perfusion flow rate of 2.0 mL/min (n = 4). No significant changes in brain/perfusate ratio of FITC-albumin as BBB non-transportable compound were observed, in the presence of Gly-Sar, indicating that Gly-Sar did not induce any BBB disruption in the present perfusion conditions. Results are expressed as the mean ± s.e.m. *N.S.*; not significant at *P* > 0.05.

**
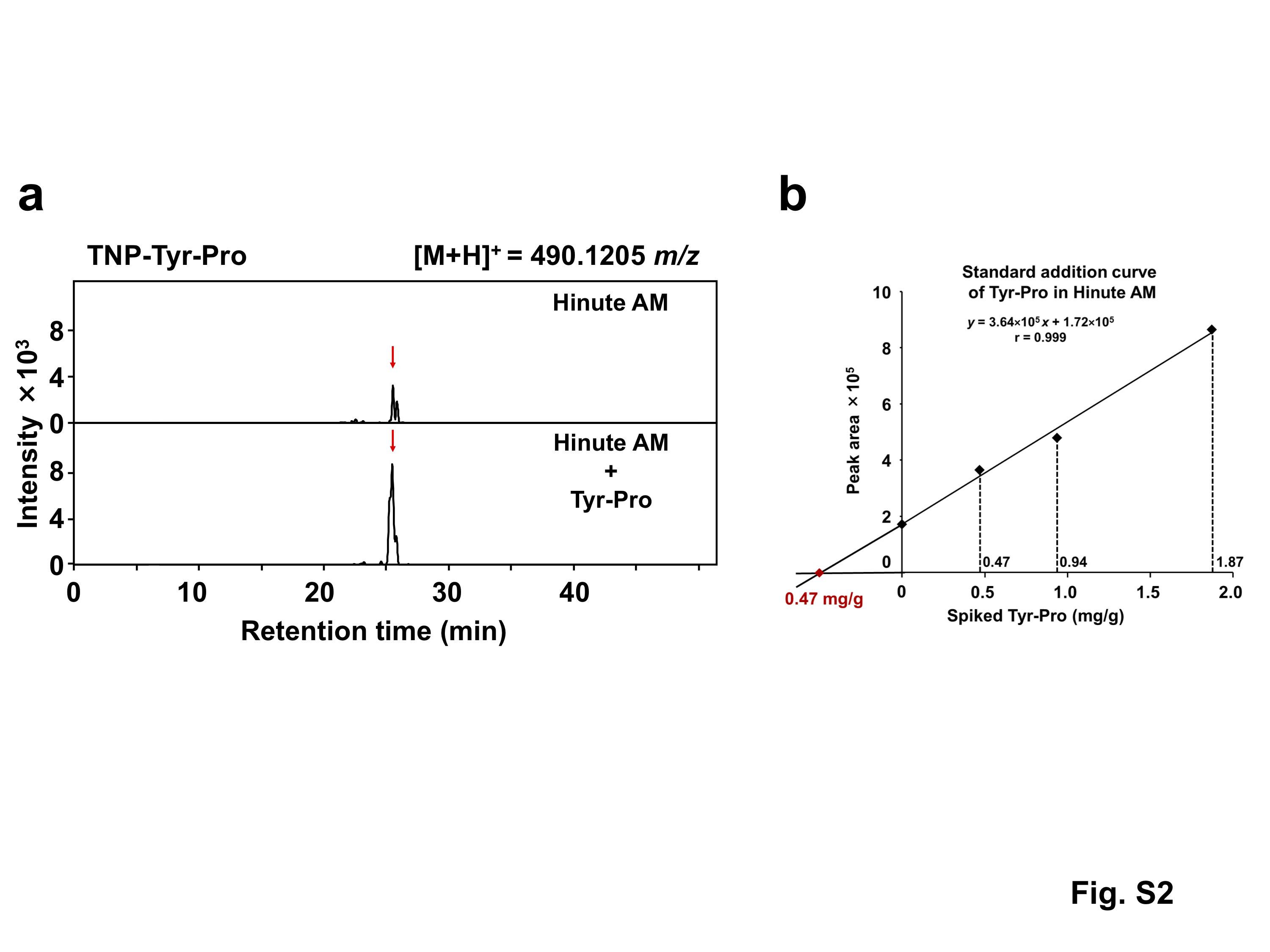
**

**Fig. S2.** **Quantitative detection of Tyr-Pro in soybean hydrolysate by TNBS-LC-TOF/MS.** (**a**) Extracted ion-chromatograms for TNP-Tyr-Pro ([M+H]^+^ = 490.1205 *m/z*, upper) from a commercially available soybean hydrolysate (Hinute AM, a product of Fuji Oil Co.) and in the hydrolysate spiked with 1.6 mg/g of Tyr-Pro, lower). The hydrolysate was dissolved in deionized water at 10 mg/mL. Tyr-Pro was successfully detected in soybean hydrolysate, indicating that Tyr-Pro is a naturally occurring dipeptide. In addition, the sequence of Tyr-Pro occurs in the sequence of soybean proteins at glycinin (89–90), glycinin (295–296), β-conglycinin α-subunit (512-513), β-conglycinin α‘-subunit (528-529), and β-conglycinin β-subunit (342-343). (**b**) Determination of Tyr-Pro in soybean hydrolysate by TNBS-aided LC-TOF/MS according to the reported standard addition method [Hahn, V. T., Kobayashi, Y., Maebuchi, M., Nakamori, T., Tanaka, M. & Matsui, T. Quantitative mass spectrometric analysis of dipeptides in protein hydrolysate by a TNBS derivatization-aided standard addition method. *Food Chem*. **190**, 345–350 (2016)]. Tyr-Pro was spiked in the hydrolysate at 0.47, 0.94, and 1.87 mg/g. According the standard addition curve (*r* = 0.999), the amount of Tyr-Pro in soybean hydrolysate was calculated to be 0.47 mg/g-hydrolysate.


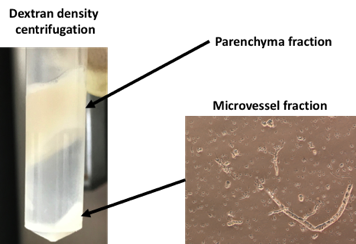


**Fig. S3. Pictures for fractionated brain parenchyma and microvessel.** A left picture clearly shows successful separation of brain parenchyma and microvessel fractions by a dextran-density centrifugation of brain homogenate. A right picture shows less contamination of parenchyma debris in the microvessel fraction.
